# Case Report: Primary endometrioid adenocarcinoma of the cervix and secondary cervical involvement by endometrial carcinoma: diagnostic pitfalls and an integrated approach based on two cases

**DOI:** 10.3389/frph.2026.1966107

**Published:** 2026-09-30

**Authors:** Lina Geng, Tianci Fan, Guanwen Gao, Yunfei Wang, Changzhong Li

**Affiliations:** 1 Department of Obstetrics and Gynecology, Peking University Shenzhen Hospital, Shenzhen, Guangdong, China; 2 Shenzhen Key Laboratory on Technology for Early Diagnosis of Major Gynecologic Diseases, Peking University Shenzhen Hospital, Shenzhen, Guangdong, China; 3 School of Medicine, Southern University of Science and Technology, Shenzhen, Guangdong, China; 4 State Key Laboratory of Reproductive Medicine and Offspring Health, Center for Reproductive Medicine, Institute of Women, Children and Reproductive Health, Shandong University, Jinan, China

**Keywords:** cervical involvement, endometrial carcinoma, immunohistochemistry, lynch syndrome, primary endometrioid adenocarcinoma of the cervix

## Abstract

**Background:**

Primary endometrioid adenocarcinoma of the cervix (PEAC) is a rare subtype of HPV-independent cervical adenocarcinoma. Due to overlapping clinical, histological, and immunophenotypic features, distinguishing PEAC from endometrioid adenocarcinoma of endometrial origin with cervical involvement remains challenging.

**Case presentation:**

We present two cases illustrating the diagnostic pitfalls in determining the primary site of endometrioid adenocarcinoma involving the cervix. The first case was ultimately diagnosed as PEAC based on comprehensive evaluation of the hysterectomy specimen, with no identifiable endometrial primary lesion. Molecular testing revealed microsatellite instability-high status and a pathogenic PMS2 germline variant associated with Lynch syndrome. The second case represented lower uterine segment endometrioid adenocarcinoma with secondary cervical involvement, which closely mimicked a cervical primary tumor. Careful assessment of tumor distribution and the relationship between uterine and cervical lesions supported an endometrial origin.

**Conclusion:**

The primary site of endometrioid adenocarcinoma involving the cervix cannot be reliably determined by morphology or immunohistochemistry alone. Accurate diagnosis requires integrated evaluation of clinical, radiological, pathological, and molecular findings, with comprehensive examination of the hysterectomy specimen—particularly assessment of the tumor epicenter, lesion distribution, and the presence of precursor lesions—remaining crucial for definitive site assignment in operable cases. The suggestive framework presented here is intended to provide a systematic, teaching-oriented approach to this challenging differential diagnosis.

## 1 Introduction

In the 2020 World Health Organization (WHO) Classification of Female Genital Tumors, cervical adenocarcinomas are categorized into two major groups based on HPV association: HPV-associated (HPVA) and non-HPV-associated (NHPVA) adenocarcinomas. NHPVA is further classified into four histological subtypes: gastric-type, clear cell, mesonephric, and endometrioid adenocarcinomas (1, 2). The diagnosis of NHPVA remains particularly challenging because of the lack of specific diagnostic biomarkers, considerable histomorphology heterogeneity, and substantial overlap with primary tumors arising elsewhere in the female genital tract. Among these, primary endometrioid adenocarcinoma of the cervix (PEAC) is an exceptionally rare subtype that is frequently misdiagnosed as endometrioid adenocarcinoma of endometrial origin with secondary cervical involvement (2–4). Such diagnostic pitfalls may result in incorrect identification of the primary tumor site, leading to inappropriate surgical staging and suboptimal treatment planning.

PEAC accounts for fewer than 5% of cervical adenocarcinomas and predominantly affects perimenopausal and postmenopausal women (5), with irregular vaginal bleeding, postcoital bleeding, and vaginal discharge being the most common presenting symptoms. However, PEAC lacks distinctive clinical features and is clinically indistinguishable from conventional cervical adenocarcinoma, making early diagnosis based on clinical presentation alone difficult (2, 6).

To date, the published literature on PEAC has been limited largely to isolated case reports, and no universally accepted diagnostic criteria or management strategy has been established. Consequently, awareness of this rare entity remains limited, contributing to frequent preoperative misdiagnosis. In this report, we present two cases illustrating the diagnostic challenges in determining the primary site of endometrioid adenocarcinoma involving the cervix. The first case was confirmed as PEAC, whereas the second case represented endometrial carcinoma with secondary cervical involvement. We describe its clinical presentation, diagnostic workup, pathological findings, and treatment course, and review the available literature to summarize the key points for differential diagnosis and clinical management. To further illustrate the diagnostic challenge, we also present a comparative case of endometrioid adenocarcinoma involving both the endometrium and cervix, in which comprehensive pathological evaluation ultimately excluded primary cervical endometrioid adenocarcinoma.

## 2 Case presentations

### 2.1 Case 1

A female patient with an unremarkable past medical and personal history underwent routine cervical screening in 2019, when liquid-based cytology revealed atypical squamous cells, cannot exclude high-grade squamous intraepithelial lesion (ASC-H). Colposcopic examination with biopsy confirmed cervical squamous intraepithelial lesion. The patient subsequently underwent loop electrosurgical excision procedure (LEEP), preoperative colposcopic examination revealed acetowhite epithelium on the ectocervix (Figure 1) and postoperative histopathological examination demonstrated cervical intraepithelial neoplasia grade 2 (CIN2) with focal CIN1. The surgical margins were negative.

**Figure 1:**
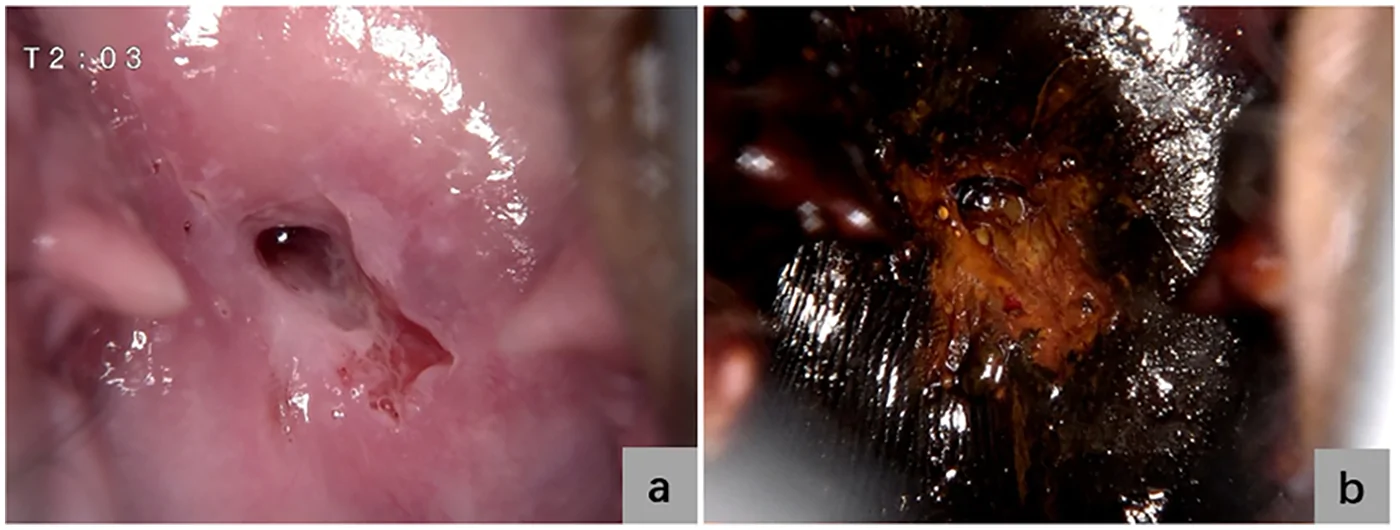
Colposcopy before LEEP: **(a)** acetic acid test showed focal thin acetowhite epithelium with sharp margins in the transformation zone (×8); **(b)** Lugol's iodine test showed a well-defined iodine-positive area matching the acetowhite lesion (×7.5).

During follow-up, HPV testing became negative after transient positivity. However, recurrent HPV58 infection was detected in 2022. Colposcopy showed a Type III transformation zone, thin pale epithelium at 6 and 12 o'clock in the vaginal fornix, and partial iodine uptake on Schiller test (Figure 2). Cervical biopsy showed low-grade squamous intraepithelial lesion (LSIL). Endocervical curettage (ECC) subsequently revealed scattered endometrioid-type glands with papillary proliferation and eosinophilic cytoplasmic changes, raising suspicion for an atypical glandular lesion.

**Figure 2:**
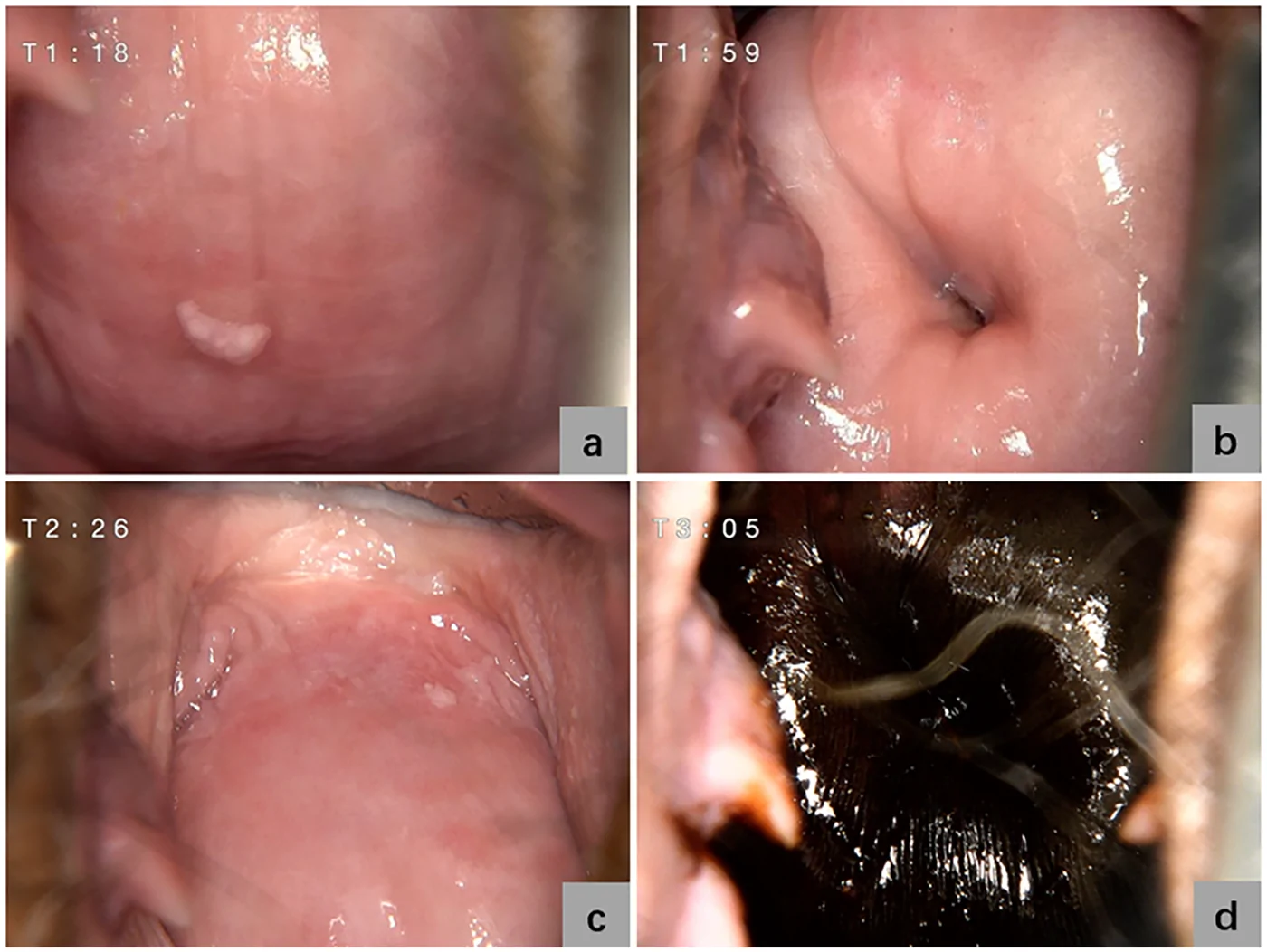
Colposcopy after LEEP: **(a)** focal thin acetowhite epithelium at 6 o'clock in vaginal fornix (×8); **(b)** normal squamous epithelium (×8); **(c)** focal thin acetowhite epithelium at 12 o'clock in vaginal fornix (×8); **(d)** iodine uptake on Lugol's test (×8).

The patient underwent diagnostic hysteroscopy on June 28, 2022. Hysteroscopic evaluation demonstrated a patent cervical canal and a 0.5 cm pedunculated endometrial polyp within the uterine cavity; bilateral tubal ostia were clearly visualized. Endometrial polypectomy with fractional curettage was subsequently performed. Histopathologic assessment demonstrated proliferative endometrium with focal polypoid hyperplasia and chronic endocervicitis with squamous metaplasia.

Six months later, persistent HPV58 infection was noted. Colposcopy showed normal squamous epithelium. Repeat ECC demonstrated complex papillary proliferation of endometrioid-type glands, suggestive of atypical glandular hyperplasia. Immunohistochemical examination showed ER positivity, patchy p16 expression, and an increased Ki-67 proliferation index. Pelvic ultrasonography and magnetic resonance imaging demonstrated a cervical mass suspicious for malignancy (Figure 3). Repeat hysteroscopy with endometrial and endocervical curettage identified endometrioid adenocarcinoma in both specimens. Immunohistochemical findings showed partial ER and PR positivity, patchy p16 expression, and a wild-type p53 staining pattern. Although a cervical primary tumor was initially favored, an endometrial origin could not be excluded.

**Figure 3:**
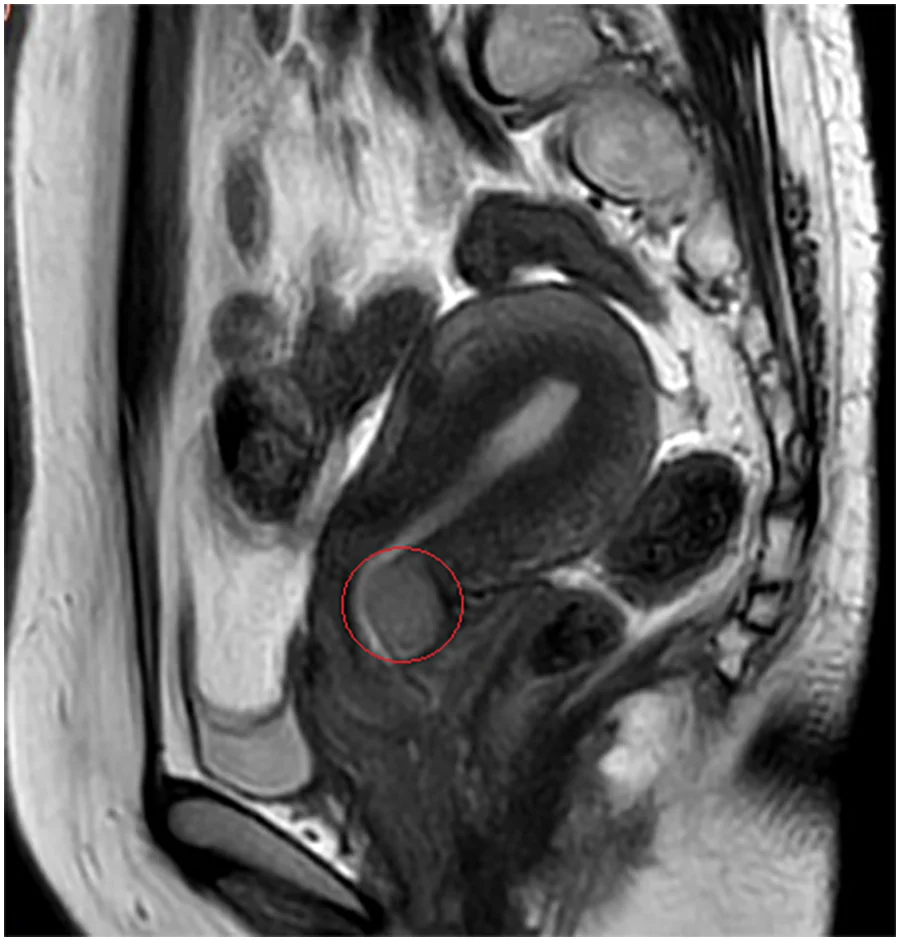
MRI showed cervical enlargement, mild posterior uterine wall endometrial thickening, and a 12 mm × 21 mm × 16 mm soft-tissue mass in the posterior cervical stroma. The red circle indicates the tumor lesion involving the lower uterine segment and extending into the cervix.

The patient subsequently underwent radical hysterectomy with bilateral salpingo-oophorectomy and pelvic and para-aortic lymphadenectomy. The tumor was predominantly centered in the cervix, without evidence of an endometrial primary lesion. Final pathology confirmed FIGO grade 1 primary endometrioid adenocarcinoma of the cervix with superficial cervical stromal invasion, lymphovascular space invasion, and presacral lymph node metastasis (1/8), all other pelvic lymph node groups and para-aortic lymph nodes were negative for malignancy, corresponding to FIGO stage IIIC1p disease.

Molecular analysis demonstrated microsatellite instability-high (MSI-H) status. Germline genetic testing of peripheral blood identified a PMS2 c.988 + 1G > A mutation, supporting a Lynch syndrome-associated tumor.

The patient received adjuvant chemoradiotherapy and remains clinically disease-free at the last follow-up.

### 2.2 Case 2

To further illustrate the principal differential diagnosis of PEAC, we present a second case of lower uterine segment endometrioid adenocarcinoma with secondary cervical involvement, which closely mimicked primary endometrioid adenocarcinoma of the cervix.

A 49-year-old woman had undergone LEEP for cervical high-grade squamous intraepithelial lesion (HSIL) approximately 10 years previously. Subsequent annual cervical cytology, HPV testing, and pelvic ultrasonography showed no significant abnormalities.

In 2024, transvaginal ultrasonography detected a solid cervical mass. Colposcopic biopsy and endocervical curettage demonstrated endometrioid adenocarcinoma (Figure 4). Immunohistochemical examination showed ER positivity, weak PR expression, patchy p16 staining, wild-type p53 expression, and a high Ki-67 proliferation index, whereas vaginal wall biopsy showed no evidence of malignancy. MRI demonstrated irregular cervical soft-tissue mass, 29 × 21 × 31 mm—concerning for malignancy.

**Figure 4:**
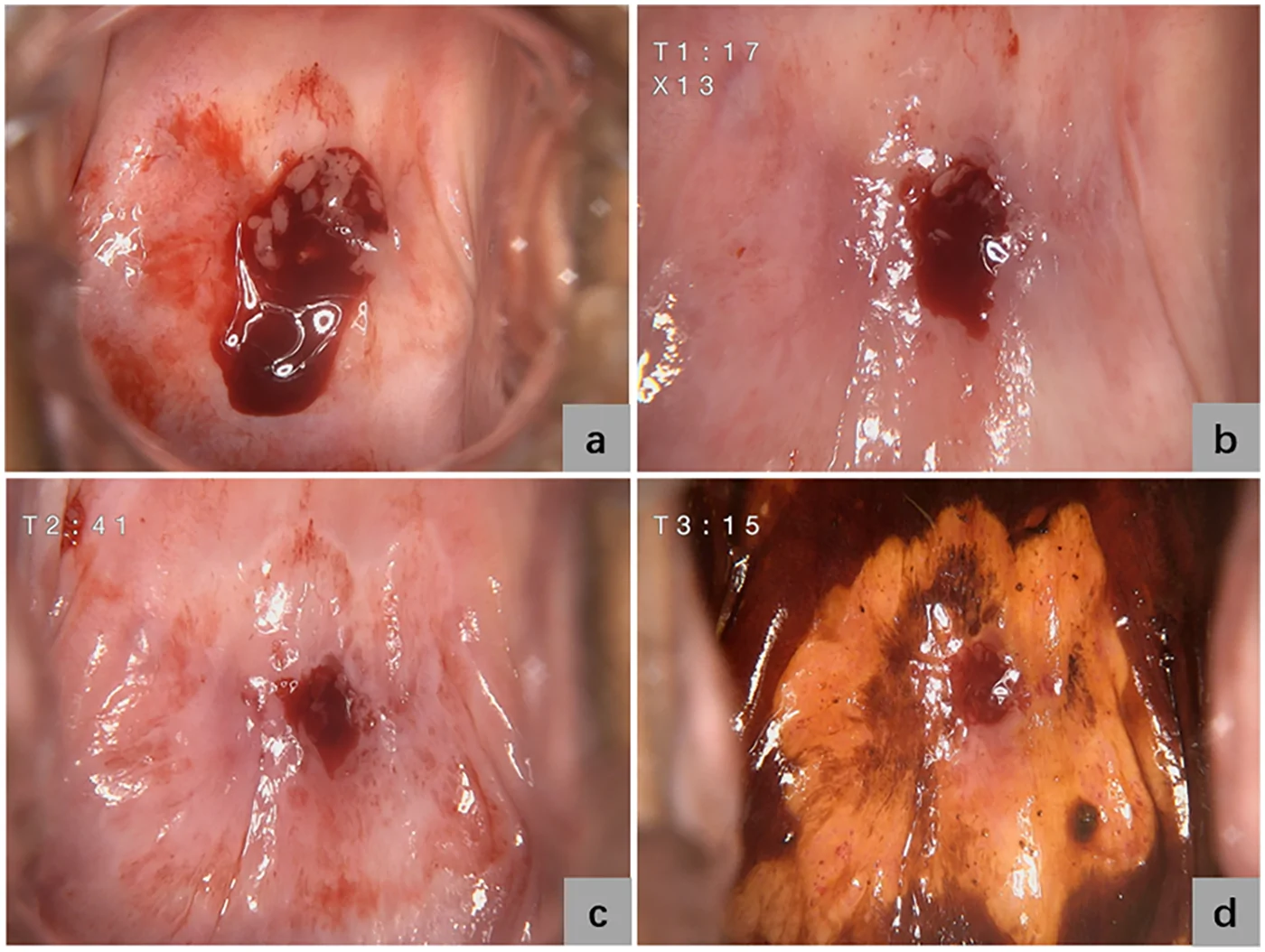
Colposcopy: **(a)** fragile tissue observed at the external cervical os (×8); **(b)** acetic acid test showed thick acetowhite epithelium (×8); **(c)** atrophic squamous epithelium (×8); **(d)** iodine partial uptake on Lugol's test (×8).

The patient subsequently underwent laparoscopic radical hysterectomy with bilateral salpingo-oophorectomy and pelvic lymphadenectomy. Histopathological examination revealed FIGO grade 1 endometrioid adenocarcinoma involving the lower uterine segment and endocervical canal, accompanied by superficial myometrial invasion (<50%). No lymph vascular space invasion, parametrial involvement, vaginal involvement, or lymph node metastasis was identified. The presence of tumor involvement extending from the lower uterine segment to the endocervical canal, together with the absence of a cervical-centered lesion, supported an endometrial origin. Immunohistochemical examination demonstrated intact mismatch repair protein expression, ER and PR positivity, patchy p16 staining, and wild-type p53 expression. The patient recovered uneventfully and remains disease-free during follow-up.

## 3 Discussion

PEAC is a rare subtype of HPV-independent cervical adenocarcinoma. Due to its considerable overlap with lower uterine segment endometrioid carcinoma in clinical presentation, morphology, and immunophenotype, accurate preoperative diagnosis remains challenging. As no universally accepted diagnostic criteria have been established, the diagnosis relies on a comprehensive assessment of clinical findings, imaging features, and pathological evaluation.

The two cases presented herein represent two clinically important diagnostic scenarios. Case 1 was ultimately confirmed as primary endometrioid adenocarcinoma of the cervix, although an endometrial origin could not be excluded preoperatively. In contrast, Case 2 represented lower uterine segment endometrioid carcinoma with secondary cervical involvement, which closely mimicked a primary cervical tumor in clinical and pathological features. These two cases demonstrate that the primary site of endometrioid adenocarcinoma involving the cervix cannot be reliably determined based solely on morphology or immunophenotype.

Gross examination may provide initial clues regarding the primary site of the tumor. Cervical primary tumors typically demonstrate a cervical-centered distribution, whereas endometrioid carcinomas of the uterine corpus are usually centered within the endometrial cavity. However, tumor location and size alone cannot reliably determine the site of origin, as secondary cervical involvement may occasionally be more extensive than the primary lesion (7). In our two cases, preoperative biopsy specimens, including cervical biopsy and endometrial sampling, showed similar morphological and immunophenotypic features, making it difficult to distinguish the tumor origin. The final diagnosis relied on comprehensive evaluation of the radical hysterectomy specimens, particularly the overall tumor distribution and the relationship between cervical and endometrial lesions. These findings further emphasize that determination of the primary site should not be based solely on limited biopsy specimens.

Immunohistochemical markers, including ER, PR, p16, and p53, often show overlapping expression patterns in these two types of adenocarcinomas and therefore cannot be used alone to determine the primary site (6, 8, 9). In both cases presented in this study, the tumors demonstrated ER and PR positivity, patchy p16 expression, and a wild-type p53 staining pattern, indicating that immunophenotypic findings should be considered supportive rather than definitive evidence for determining tumor origin. In diagnostically challenging cases, particularly those with poorly differentiated morphology, HPV testing and molecular classification may provide additional supportive information in selected diagnostically challenging cases.

Table 1 summarizes a suggestive, teaching-oriented diagnostic framework integrating clinical, radiological, pathological, immunohistochemical, and molecular findings. This framework is derived from the present cases and available literature and is intended to systematize diagnostic reasoning rather than to serve as a validated clinical decision rule or a new diagnostic standard. Based on the findings of the present study and previous reports, a practical diagnostic approach can be proposed for cases of endometrioid adenocarcinoma involving the cervix (1–4, 10, 11). First, clinical presentation and imaging findings should be carefully evaluated to identify the tumor epicenter and determine whether the lesion is primarily cervical or endometrial in origin. Second, cervical biopsy and endometrial sampling should be performed to assess the extent and distribution of the lesions at both sites. Third, immunohistochemical analysis can be used to exclude HPV-associated cervical adenocarcinoma and other specific histological subtypes. Finally, comprehensive evaluation of the entire surgical specimen remains essential, with particular attention to tumor distribution, the relationship between cervical and endometrial lesions, surrounding tissue changes, and the presence of *in situ* or precursor lesions. The primary site should ultimately be determined through integrated clinicopathological correlation and, whenever feasible, multidisciplinary discussion. This approach should be applied flexibly in the context of available diagnostic resources and individual clinical circumstances.

**Table 1:** Suggestive diagnostic framework for distinguishing primary endometrioid adenocarcinoma of the cervix from endometrial carcinoma with secondary cervical involvement.

Diagnostic modality	Key findings suggestive of PEAC	Key findings suggestive of endometrial origin	Major limitations
Clinical presentation	Predominant cervical symptoms, including vaginal bleeding, watery discharge, or an abnormal cervical lesion on examination	Symptoms or history suggestive of an endometrial lesion; cervical involvement may be secondary	Clinical manifestations frequently overlap and are nonspecific
Imaging (MRI/ultrasound)	Tumor epicenter in the cervix; cervical stromal expansion; predominant cervical mass	Tumor centered in the endometrial cavity or lower uterine segment, with extension into the cervix	Large tumors and extensive secondary cervical involvement may obscure the true tumor epicenter
Cervical biopsy and endometrial sampling	Endometrioid adenocarcinoma identified in a cervical specimen, particularly when endometrial sampling is negative or non-diagnostic	Endometrioid carcinoma identified in endometrial sampling, with cervical biopsy showing similar morphology	Limited sampling may not reflect the site of origin; identical morphology may be present at both sites
Gross examination of surgical specimen	Cervical-centered tumor with no dominant endometrial cavity lesion	Dominant endometrial cavity/lower uterine segment tumor extending into the endocervix	Not available in patients managed non-surgically; extensive tumors can distort anatomic relationships
Histomorphology	Endometrioid morphology in a cervical-centered lesion; possible cervical precursor lesion, if present	Endometrioid morphology associated with a dominant endometrial lesion; possible endometrial precursor lesion	Morphological overlap is substantial; precursor lesions may be absent, obscured, or unsampled
Immunohistochemistry	ER/PR positivity, patchy p16 expression, and wild-type p53 pattern may support an endometrioid phenotype but are not site-specific	Similar ER/PR positivity, patchy p16 expression, and wild-type p53 pattern may be observed	Considerable immunophenotypic overlap; no single marker reliably establishes the primary site
HPV testing	Negative high-risk HPV testing supports an HPV-independent cervical tumor after exclusion of HPV-associated adenocarcinoma	Negative HPV testing may also be observed in endometrial carcinoma	HPV negativity does not distinguish PEAC from endometrial carcinoma
MMR and molecular testing	MMR deficiency, MSI-high status, or other molecular alterations may support tumor characterization and identify hereditary predisposition	Molecular classification may support endometrial carcinoma risk stratification and treatment planning	Molecular findings generally do not independently establish anatomic site of origin; availability may be limited
Integrated clinicopathological assessment	Concordant cervical-centered clinical, imaging, and surgical-pathology findings	Concordant endometrial-centered clinical, imaging, and surgical-pathology findings	Requires multidisciplinary interpretation; not prospectively validated

This framework has important limitations. It is based on two illustrative cases and a narrative synthesis of the available literature, and it has not undergone prospective or external validation. Therefore, it should not be regarded as a formal diagnostic rule. Its application may be constrained by sampling error in cervical biopsy or endometrial curettage specimens, overlapping morphological and immunophenotypic features, and limited access to high-quality imaging, HPV testing, or molecular assays in different practice settings. In cases with discordant clinical, radiological, and pathological findings, multidisciplinary review and additional targeted sampling, when feasible, may be helpful. However, in operable cases, definitive assignment of the primary site may still depend on comprehensive examination of the hysterectomy specimen. Thus, the framework is intended primarily as a practical teaching tool to guide systematic diagnostic thinking.

It is important to emphasize that when preoperative pathological findings are discordant with clinical or radiological features, diagnosis should not be based solely on limited biopsy specimens. Misinterpretation of the primary site may result in inaccurate staging and inappropriate initial treatment planning. For example, when cervical biopsy demonstrates endometrioid adenocarcinoma but imaging suggests involvement of the endometrial cavity, an endometrial origin should be carefully considered and further evaluated. Accurate determination of the primary site is therefore critical, as cervical and endometrial carcinomas differ substantially in FIGO staging, surgical management, lymph node assessment, and indications for adjuvant therapy.

The therapeutic consequences of an incorrect assignment of the primary site should also be considered. Although treatment strategies may overlap in selected patients with advanced disease, cervical and endometrial carcinomas are managed according to distinct treatment paradigms. For a cervical primary, treatment is generally determined by cervical cancer stage and may include radical surgery for selected early-stage tumors or definitive concurrent chemoradiotherapy with brachytherapy for locally advanced disease. In contrast, endometrial carcinoma is typically managed by total hysterectomy with bilateral salpingo-oophorectomy and surgical staging, followed by risk-adapted adjuvant therapy based on factors such as histologic subtype, grade, myometrial invasion, lymphovascular space invasion, nodal status, and molecular classification. Consequently, misclassification may lead to an inappropriate treatment sequence or surgical procedure, inadequate or excessive lymph node assessment, inappropriate radiotherapy field design, omission or inappropriate use of brachytherapy, and inaccurate prognostic counseling and surveillance planning. Therefore, correct identification of the primary site is essential not only for diagnostic accuracy but also for individualized therapeutic decision-making.

Furthermore, with the increasing application of molecular pathology in gynecologic oncology, MMR protein assessment and genetic evaluation have become important components of individualized patient management. In our first case, the tumor demonstrated an MSI-high phenotype, and germline testing identified a PMS2 pathogenic variant, supporting a Lynch syndrome-associated carcinoma. These findings highlight the importance of genetic counseling and germline testing in patients with MMR deficiency or suspected hereditary predisposition, which may facilitate personalized management and provide relevant information for cancer risk assessment and surveillance of affected family members (12).

Together, these cases highlight that accurate classification of endometrioid adenocarcinoma involving the cervix requires integration of clinical, radiological, pathological, and molecular findings, with comprehensive examination of the surgical specimen remaining essential for determining tumor origin.

## Data Availability

The original contributions presented in the study are included in the article/Supplementary Material, further inquiries can be directed to the corresponding author.
